# How aging causes osteoarthritis: an evolutionary physiology perspective

**DOI:** 10.1016/j.joca.2025.05.001

**Published:** 2025-05-15

**Authors:** David Gems

**Affiliations:** Institute of Healthy Ageing, and Research Department of Genetics, Evolution and Environment, University College London, London WC1E 6BT, UK

**Keywords:** antagonistic pleiotropy, chondrocyte, evolutionary medicine, hyperfunction, osteoarthritis, programmatic theory

## Abstract

Late-life diseases result from the poorly understood process of senescence (aging), that is largely genetically determined. According to a recently proposed evolutionary physiology-based account, the multifactorial model, senescence is largely caused by evolved but non-adaptive programmatic mechanisms specified by the wild-type (i.e. normal) genome. These act together with disruptions to wild-type function (due e.g. to infectious pathogens, mechanical injury and malnutrition) in a variety of combinations to generate diverse late-life diseases. Here the utility of this model is explored by testing its capacity to provide an account of one complex, late-life disease, osteoarthritis (OA), and a framework for understanding OA etiology suggested. In this framework, the core OA disease mechanism is a futile endochondral ossification quasi-program (non-adaptive developmental program), in which hypertrophic articular chondrocytes alter joint architecture. Programmatic changes prime chondrocytes for quasi-program activation, which can be triggered by secondary causes of OA (e.g. joint mechanical injury). A suggested evolutionary cause of this priming, involving antagonistic pleiotropy, is selection to maximize early life tissue repair benefits at the expense of late-life programmatic costs.

## Introduction

“The physician is constantly referring to the biologist for a scientific basis for geriatrics, and finding that it is not there” (Comfort, 1979)

The biological process of senescence (aging) remains poorly defined and, consequently, our understanding of late-life disease etiologies remains far from complete. Arguably, the main purpose of biogerontology, the study of the biology of aging, is to provide an account of the mechanisms of senescence in terms of general principles, that can guide research on late-life disease (Comfort, 1979). However, thus far such accounts have remained fragmentary, and with only limited capacity to do so (Gems and de Magalhães, 2021; Gems et al., 2024a).

However, recent years have seen the emergence of a promising evolutionary physiology-based conceptual framework that combines evolutionary and proximate mechanisms to explain the causes of aging. This framework includes the evolutionary theory of aging (Arnold and Rose, 2023; Williams, 1957), the hyperfunction (or developmental) theory of aging (Blagosklonny, 2006; de Magalhães and Church, 2005), among other programmatic mechanisms (Gems and Kern, 2024), and a multifactorial model of the origins of late-life diseases (Gems, 2022).

In this article, the capacity of the multifactorial model to explain a test-case complex late-life disease, osteoarthritis, is investigated. To do this, a conceptual research approach has been used, where an existing theory is tested by examining and repurposing existing published findings (Blagosklonny and Pardee, 2002). The result is a proposed account of the multifactorial etiology of osteoarthritis in terms of general biogerontological principles. This work endeavours to bridge the conceptual gap between biogerontology and rheumatology, in a manner in which the former more usefully informs the latter.

### The multifactorial model

**Figure 1:**
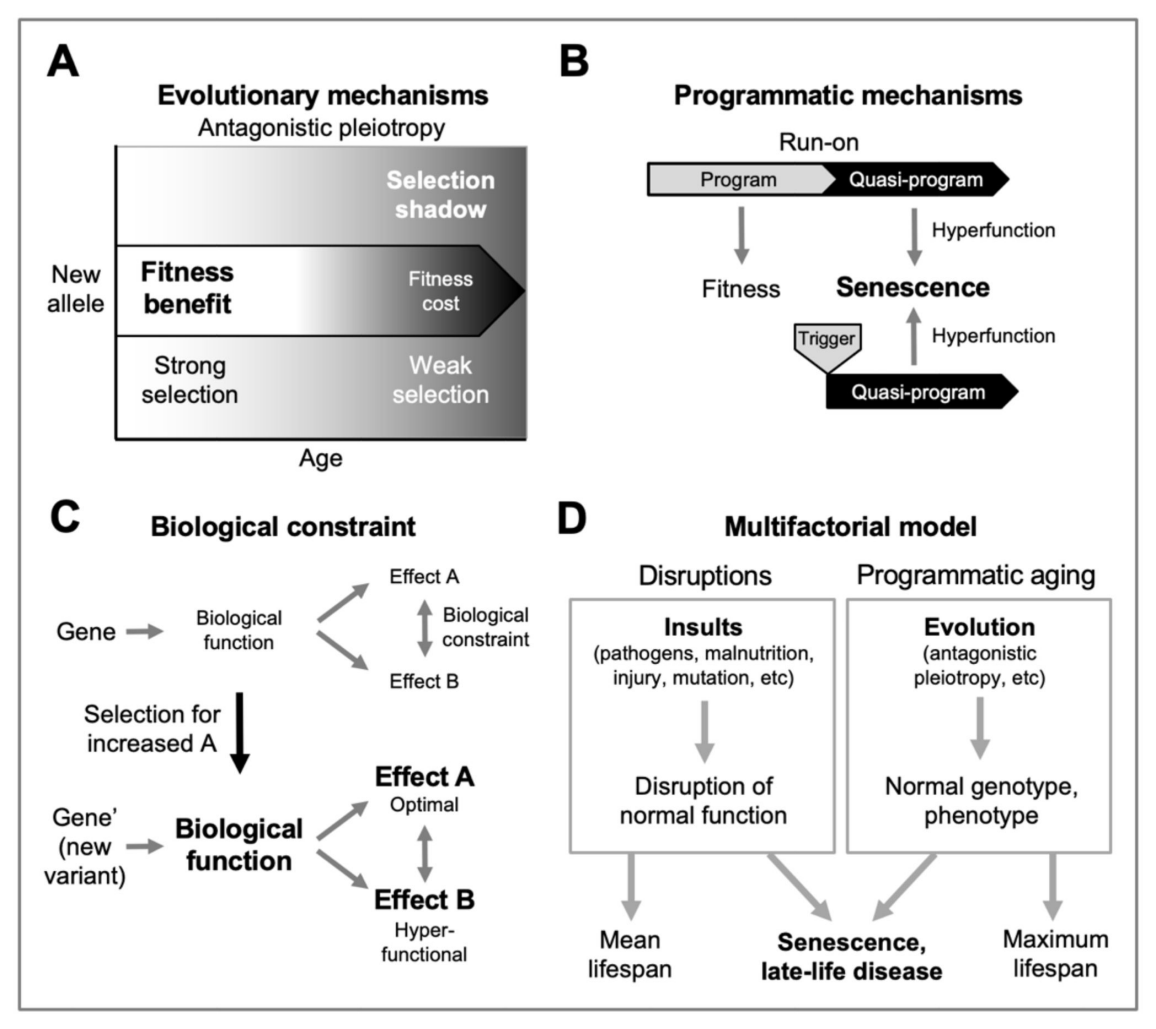
The multifactorial model and its constituent concepts. A general evolutionary medicine model. A, Antagonistic pleiotropy. A new allele that confers a fitness benefit in early life but a fitness cost (e.g. increased pathology) in later life may cause a net benefit in overall fitness due to the selection shadow (Williams, 1957). B, Examples of programmatic mechanisms of aging. Quasi-programs that contribute to senescence involve non-adaptive action of wild-type biological programs (Blagosklonny, 2006), and may arise due to futile run-on of wild-type programs (as in presbyopia) (Strenk et al., 2005), or they may be triggered by other events (as in rheumatoid arthritis) (Kern and Stebbing, 2023). C, Biological constraint as a cause of antagonistic pleiotropy. Where two traits, A and B, are coupled due to biological constraint, selection for an increase in A results in a non-adaptive increase in B, which in this case is hyperfunctional (i.e. pathogenic). Likewise, selection for reduced A can result in a non-adaptive reduction in B that is hypofunctional (also pathogenic) (Gems and Kern, 2024). D, Multifactorial model, simplified scheme (Gems, 2022). This depicts the multifactorial etiology typical of late-life diseases as arising from two broad categories of cause: disruptions to normal function (e.g. infectious pathogens, mechanical injury, mutation) (left), and programmatic consequences of wild-type genotype (right), whose pathogenicity originates in the evolutionary process. Variation in disruptions experienced contributes to inter-individual differences in lifespan, and mean lifespan. By contrast, programmatic aging sets maximum lifespan; for example, the longer maximum lifespan of humans compared to chimpanzees is a function of the normal genome, and programmatic mechanisms, not disruptions.

The foundation of the multifactorial model is the evolutionary theory of aging. The maximum lifespan of different mammalian species ranges from ~3 years in small rodents such as mice to over 200 years in bowhead whales (George et al., 1999). From this it is evident that aging rate is largely a function of the normal (wild-type) genome, as shaped by evolution. Senescence is not an adaptation, but rather a non-adaptive by-product of the evolutionary process (Arnold and Rose, 2023). Here a major determinant is the declining force of natural selection with increasing age (Medawar, 1952), sometimes referred to as the late-life selection shadow. Many genes are pleiotropic (affecting multiple characteristics), and a given new allele can result in phenotypic changes that, in different ways, both increases and reduces fitness - it exhibits antagonistic pleiotropy. Where beneficial effects occur earlier in life but deleterious ones much later, natural selection may favor the new allele, though it promotes pathology (senescence) as well as fitness (Williams, 1957) (Figure 1A).

Though evolutionary theory largely explains why aging exists, it leaves undefined the proximate mechanisms of senescence, including the precise causes of late-life disease. Such an explanation in terms of evolutionary physiology (Arnold and Rose, 2023) is what the new programmatic theories aim to provide. According to these, senescent changes are specified by the genome, as earlier life traits are, but differ from them in being non-adaptive. One proposed form of programmatic aging involves developmental functions that in later life are executed in a futile and pathogenic fashion (de Magalhães and Church, 2005). These are programmed in the mechanistic sense, but not the adaptive sense, or *quasi-programmed* (Blagosklonny, 2006).

According to this view, cells and tissues contributing to pathology often exhibit *hyperfunction*, i.e. a level of activity that is in excess of what is optimal to maintain health (Blagosklonny, 2006) (Figure 1B) rather than, as is more traditionally assumed, breakdown of cellular function (e.g. molecular damage, disruptions to organelles). Such quasi-programs may result from run-on of ontogenetic functions (Blagosklonny, 2006; de la Guardia et al., 2016; de Magalhães and Church, 2005) or, perhaps more plausibly, from triggering in later-life of adult, maturo-developmental programs (e.g. of tissue homeostasis, wound-healing and reproduction) (Gems et al., 2024b; Kern and Stebbing, 2023).

A previously influential theory of aging is that it is the result of molecular damage, particularly reactive oxygen species (ROS) generated as a by-product of mitochondrial respiration (Beckman and Ames, 1998). A proposition of programmatic theory is that the increased levels of oxidative damage observed in later life are more a consequence than a cause of senescent changes (Blagosklonny, 2008). In line with this, antioxidant treatment has little effect on senescence, including osteoarthritis (Loeser, 2017).

Programmatic theory argues that genes frequently exhibit antagonistic pleiotropy due to the ubiquity of biological constraint, arising in particular from the interconnected nature of biological functions (Acerenza, 2016; Gems and Kern, 2024). Thus, selection for a change in one trait can result in a non-selected change in a coupled trait, which may promote pathology through hyperfunction or hypofunction (Figure 1C).

Though programmatic theory goes some way towards an account of aging in terms of evolutionary physiology, it is insufficient to provide detailed explanations of late-life disease etiology. To overcome this, the multifactorial model was devised, by integrating programmatic theory with the earlier four models theory of late-life disease causation proposed by the gerontologist V.M. Dilman (Dilman, 1994; Gems, 2022). In outline, this model is as follows.

A typical and complex feature of late-life diseases is that they are multifactorial in etiology. The multifactorial model identifies two broad categories of etiology that combine in diverse ways to generate late-life disease (Gems, 2022) (Figure 1D). The first category, *disruptions*, includes the diverse insults that cause most diseases of earlier life - infectious pathogens, sub-optimal nutrition, mutation (somatic and inherited), mechanical injury, and so on. Here, in each case, normal biological function is disrupted by such factors, leading to disease. The second category includes programmatic changes, consequences of the normal, wild-type genome, whose pathogenic behavior is a consequence of the evolutionary process.

As an illustration of interpretation of disease etiology in terms of the multifactorial model, consider sudden acute respiratory syndrome (SARS), a frequent cause of death during the recent COVID-19 pandemic. This largely afflicted the elderly, due to age-related hyperfunction of the innate immune system, of likely programmatic origin (Blagosklonny, 2020). SARS occurred when infection with the coronavirus SARS-CoV-2 triggered a lethal immunological quasi-program (cytokine storm). Thus, here SARS is a multifactorial disorder, due to combined effects of programmatic changes (immunosenescence) and a disruption (a virus).

### Osteoarthritis is a function of biological age

Osteoarthritis (OA) is a slow, progressive, degenerative, multifactorial disease of joints that particularly afflicts the knee and hip joints, and those of the lower back and the neck (Allen et al., 2022). The affected joints can become stiff and painful, seriously affecting around 10% of men and 18% of women over 60 (Glyn-Jones et al., 2015). Reflecting the numerous theories of aging, there are many theories about the causes of OA (Aigner et al., 2004). These include accumulated mechanical wear-and-tear to joint cartilage (Radin et al., 1991); glycation of cartilage collagen (Liu et al., 2024); chondrocyte apoptosis due to mitochondrial dysfunction (Blanco et al., 2011; Loeser, 2011) or deficiency in the unfolded protein response (Huang et al., 2022); accumulation of senescent chondrocytes (Jeon et al., 2018; McCulloch et al., 2017) caused by DNA damage (Coryell et al., 2021; Henrotin et al., 2005; Martin et al., 2004; Yudoh et al., 2005); epigenetic changes (Tangredi and Lawler, 2020); deficiency in autophagy (Lotz and Caramés, 2011; Luo et al., 2019); inflammaging (Motta et al., 2023); the microbiome (Arora et al., 2021; Favazzo et al., 2020); and the hallmarks of aging (Mobasheri et al., 2015).

Comparing these theories against the multifactorial model, two particular and related short-comings stand out: an over-emphasis on disruption-type explanations, and a neglect of the question of why OA only emerges in later life. Disruption-type theories of aging sometimes explain the delayed onset of senescence in terms of slow, time-dependent cumulative processes, as in the gradual oxidation over time of metal, for example as slow accumulation of DNA damage. Yet OA onset is less a function of time than of the proportion of the overall life history that has been played out, as is evident from the comparative biology of this disease.

OA occurs widely among mammals, including lions, hyenas, bears, camels, elephants and dolphins, which in the latter particularly affects the humeral trochlea at the base of the flipper (Föllmi et al., 2007; Greer et al., 1977; Nganvongpanit et al., 2017). Longer-lived species develop OA later than shorter-lived species. Comparing approximate estimates for age of onset of OA and mean lifespan in selected species, one sees mouse (*Mus musculus*), onset: 9-12 months (Poole et al., 2010), lifespan: 12-18 months; rabbit (*Oryctolagus cuniculus*), onset: 6-9 years (Arzi et al., 2012), lifespan: 8-12 years; rhesus monkey (*Macaca mulatta*), onset: 16-20 years (DeRousseau, 1985; Duncan et al., 2012), lifespan: 25-30 years; human (*Homo sapiens*), onset: 50-60 years, lifespan: 80-85 years. Thus, the timing of OA onset approximately scales to lifespan across mammalian species that exhibit the disease.

The timing of OA is therefore a function of biological age (i.e. relative age), not chronological age. Such scaling to lifespan of time of onset is typical of many diseases of aging, illustrating how late-life disease is rooted in an underlying aging process that occurs at very different rates. These differences are specified by the wild-type genome and, ultimately, by the evolutionary process. Thus, the primary causes of OA and main determinants of its timing of onset are almost certainly programmatic, rather than disruption-based. Ultimately, OA, like other afflictions of old age, is a disease of evolution, fully explicable only in terms of evolutionary medicine (Brüne and Schiefenhövel, 2019; Nesse and Williams, 1994). Yet having said this, one disruption theory of OA is particularly well supported.

### Osteoarthritis as a triggered quasi-program

The wear-and-tear theory argues that in joints, as in moving parts of machinery, the physical stress of repeated use leads to eventual wearing out (Radin et al., 1991). This views OA as a disease of mechanical senescence. Supporting this, OA risk is increased by injury to joints, and also by imperfections in joint structure due to developmental or genetic abnormalities (Glyn-Jones et al., 2015). Similarly, obesity, which increases stress on weight-bearing joints, increases the likelihood of developing OA, particularly of the knee, where risk is increased three-fold (Blagojevic et al., 2010). But again, as with other theories, where the wear-and-tear theory falls short is in explaining why OA develops only later in life. For example, intra-articular knee fracture is 3-4-times more likely to trigger OA development after the age of 50 (Honkonen, 1995; Stevens et al., 2001; Volpin et al., 1990).

**Figure 2:**
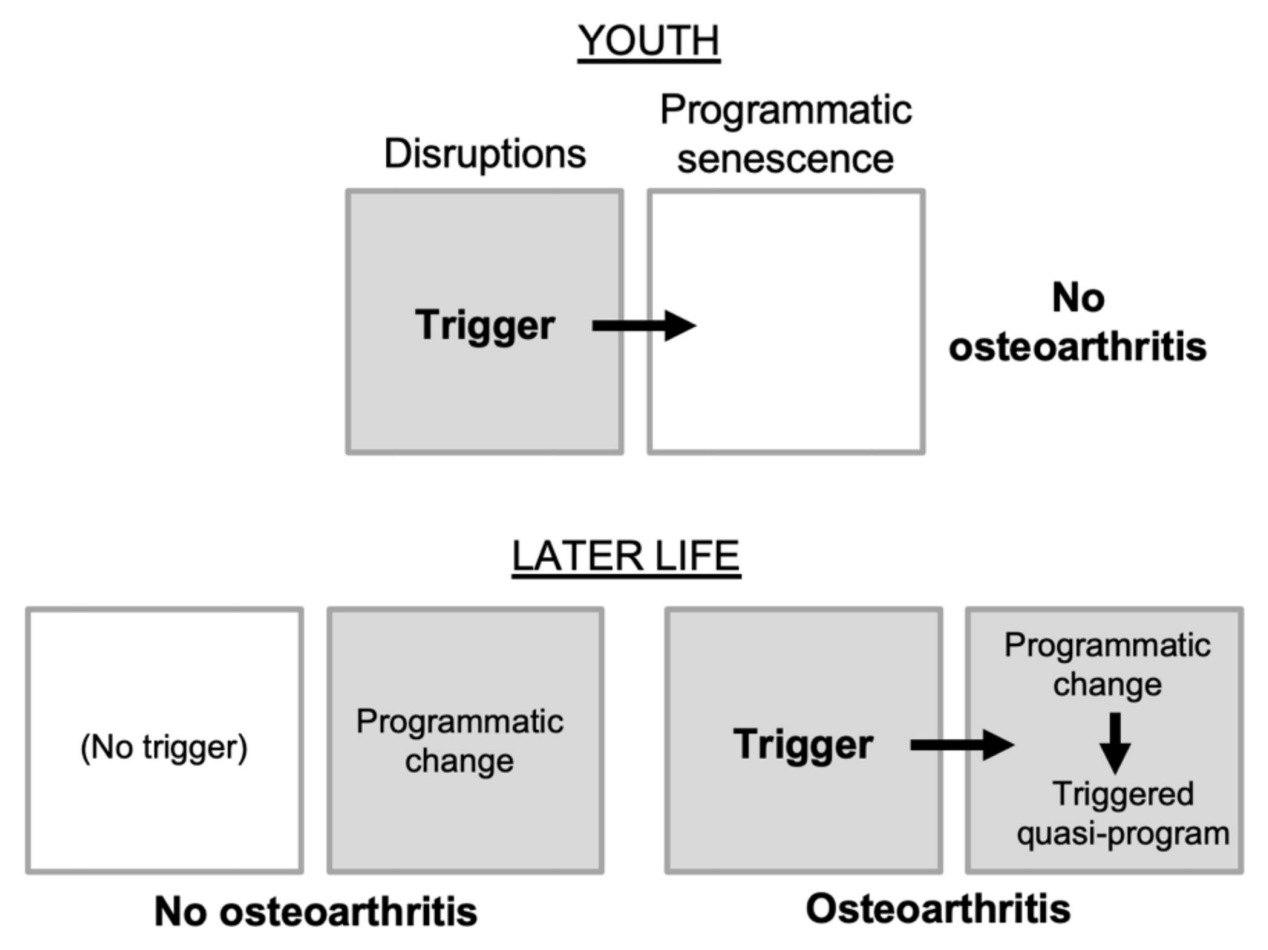
Osteoarthritis as a triggered quasi-program. Based on the multifactorial model (Figure 1D); grey fill indicates active etiology. Top, in youth, disruptions (e.g. joint injury) do not trigger OA development (analogous to pulling the trigger of an unloaded gun). Bottom left, in later life, programmatic changes occur that predispose to OA (analogous to the loading of a gun), but may be insufficient to cause disease. Bottom right, disruptions trigger OA development where programmatic changes create a predisposition. By the same principle, infection with coronavirus SARS-CoV-2 triggers fatal SARS far more frequently in the elderly than in the young (Figure 1D).

Here the multifactorial model provides a hypothesis: that OA is the result of a triggered quasi-program (Figure 1B). In this form of etiology, programmatic changes in later life create conditions in which quasi-program triggering becomes possible. According to this hypothesis, mechanical stress to joints acts as a trigger to initiate OA only once programmatic changes have occurred. The causal relationship between trigger and quasi-program resembles that in a gun: though pulling the trigger is the cause of the gun firing, the major cause of the gun shot is the cartridge. Thus, the process of aging effectively loads the OA gun, such that triggers can lead to it firing. In line with this, stress on joints in later life may trigger disease onset, or damage from injuries to joints incurred early in life can lie latent for many years before leading in later life to OA (Juhakoski et al., 2009; Toivanen et al., 2010). In the latter case, the trigger was, as it were, being pulled earlier in life without consequence prior to quasi-program loading (Figure 2).

Accounts of OA distinguish two forms: primary and secondary. Primary OA is sometimes described as idiopathic (in other words, a disease of unknown cause). Secondary OA is a consequence of known causes, such as joint injury, gout, diabetes or obesity (Glyn-Jones et al., 2015). Arguably, primary OA is that arising from programmatic aging, while secondary arthritis involves additional accelerants of programmatic aging, particularly disease triggers, caused by disruptions or other quasi-programs.

### Osteoarthritis as a programmatic disease

Next let us consider the possible role of programmatic changes in OA. The programmatic theory predicts (i) that destructive programmatic mechanisms linked in particular to tissue-level homeostasis emerge in joints in later life; and (ii) that these will involve triggered programs, leading to destructive developmental and morphogenetic changes. Several features of OA are broadly consistent with this scenario. First, cell types that control tissue homeostasis in joints (particularly chondrocytes) show hyperfunctional and quasi-programmed changes in later life (described below). Second, OA does involve complex and concerted developmental and morphogenetic changes.

**Figure 3:**
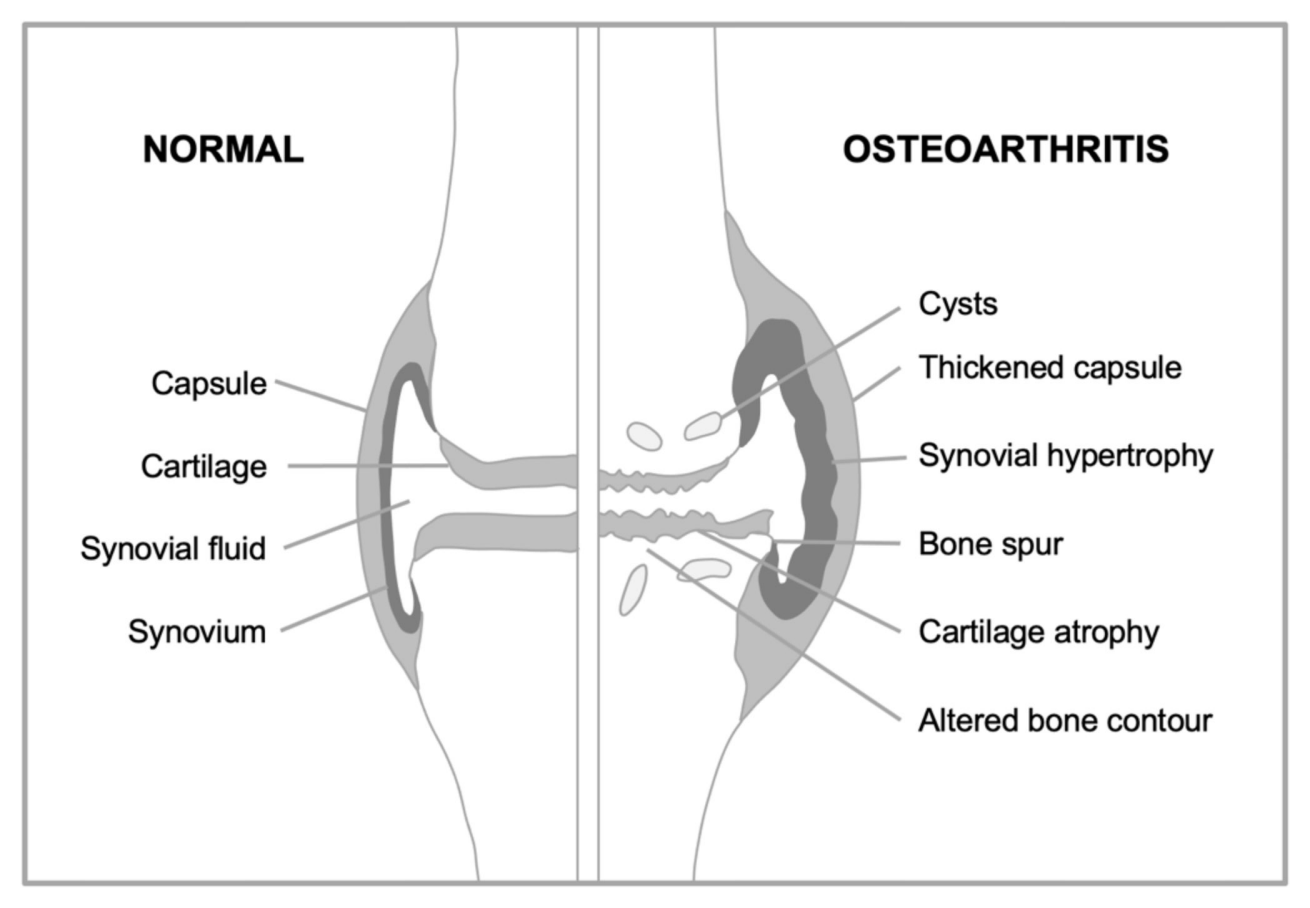
Developmental and morphogenetic changes occurring during osteoarthritis. Schematic representation of a knee joint that is normal (left), and with osteoarthritis (right). Major changes in the disease include a loss of the cartilage that allows smooth articulation of bones during joint movement, and overgrowth of new bone.

Consider first how overall joint structure changes during OA (Figure 3). The surfaces of the two opposing bones are protected by a layer of smooth, tough hyaline cartilage, which is bathed in synovial fluid. The joint is surrounded by a joint capsule, lined by the synovial membrane (or synovium) which secretes into the synovial fluid lubricants such as hyaluronan and lubricin. In OA, the articular cartilage atrophies, while the synovium and capsule become hypertrophied. But the critical anatomical changes take place at the boundary between the cartilage and underlying bone (the subchondral bone). Here, futile activation of a bone formation (endochondral ossification) program takes place (Dreier, 2010), leading to loss of cartilage and changes in subchondral bone, including development of bone marrow pockets, a blood supply, and fluid-filled subchondral cysts (Glyn-Jones et al., 2015). Subchondral bone hypertrophy may also contribute to formation of bone spurs (osteophytes), that can restrict joint movement and cause pain (Felson et al., 2005). Thus, OA is a disease involving complex developmental and morphogenetic changes in joints, starting in later life.

A prediction of programmatic theory is that tissue-level changes leading to OA will result from hyperfunctional changes to cells. Four main cell types contribute to tissue homeostasis within the joint: osteocytes in the bone, synoviocytes (fibroblast- and macrophage-like) in the synovium and, particularly, chondrocytes that generate and maintain cartilage. Thus, key to OA is understanding how and why chondrocyte function changes in later life. Consequently, much research on OA has focused on age changes in chondrocytes and it is here, arguably, that disruption-based explanations are particularly inadequate.

### The senescent chondrocyte hypothesis

An influential disruption-type account of aging is that it is caused by molecular damage accumulation. In line with this, it has been proposed that cellular stress and molecular damage cause pathogenic changes in chondrocytes. One scenario is that this causes cell death, promoting OA. Apoptotic and dead chondrocytes have been observed in degenerating cartilage in OA (Blanco et al., 1998; Hashimoto et al., 1998), and autophagy has been proposed as a mechanism that protects against chondrocyte apoptosis that otherwise promotes OA (Luo et al., 2019). An alternative view is that aging chondrocytes show aberrant behavior, destroying articular cartilage, due e.g. to disruption of cellular signaling by elevated ROS levels (Loeser, 2017). More recently, such changes have been interpreted as DNA damage-induced cellular senescence (Coryell et al., 2021), suggesting that accumulation of senescent cells causes OA (Jeon et al., 2018; McCulloch et al., 2017). But what exactly is cellular senescence?

During cellular senescence, cells leave the cell cycle and undergo a major differentiative change, becoming hypertrophic and hypersecretory. Senescent cells accumulate during aging, and exert pathogenic effects due mainly to hypersecretion (Birch and Gil, 2020). The senescence-associated secretory phenotype (SASP) includes a variety of factors that alter the local tissue microenvironment in diverse ways, including proteases, growth factors and inflammatory cytokines (Basisty et al., 2020). Particularly striking was the finding that in aging mice elimination of senescent cells delayed onset of many late-life diseases, including cancer, atherosclerosis and kidney disease, and also markedly extended lifespan (Baker et al., 2016; Childs et al., 2016).

Drawing on these advances a cellular senescence theory of OA emerged, with good evidential support. In later life, chondrocytes from human articular cartilage show increased levels of two markers of cellular senescence, lysosomal β-galactosidase (senescence-associated β-galactosidase, or SA-β-Gal), and p16^INK4a^ (p16) mRNA (Diekman et al., 2018; Martin and Buckwalter, 2003). In mouse cartilage, p16 mRNA also increases with age (Diekman et al., 2018). If senescent chondrocyte accumulation is a cause of OA, then their elimination from osteoarthritic joints using senolytic drugs might ameliorate the disease (Coryell et al., 2021). Supporting this, in a mouse model of injury-induced OA, clearance of p16-positive cells in the p16-3MR transgenic mouse, or intra-articular injections of the senolytic compound UBX0101, reduced senescent chondrocyte numbers and SASP levels and slowed progression of OA (Jeon et al., 2017).

But why do senescent chondrocytes accumulate? More broadly, the causes of cellular senescence leading to senescent cell accumulation *in vivo* remain unclear (Wiley and Campisi, 2021). One suggestion is that chondrocyte senescence is a consequence of DNA damage and cellular stress (Coryell et al., 2021). An alternative possibility, discussed next, is that such late-life alterations in chondrocyte function are a consequence of developmental change.

### Reinterpreting cellular senescence

In recent years, a new perspective on cellular senescence has emerged (Davan-Wetton et al., 2021; Elder and Emmerson, 2020; Gems and Kern, 2022; Kuilman et al., 2010; Paramos-de-Carvalho et al., 2021). A long-standing puzzle was why senescent cells should behave in so actively pathogenic a manner, particularly though SASP production. This was solved by the discovery that senescent cells have a developmental function, particularly in tissue remodeling (Yun, 2018). For example, senescent fibroblasts contribute to wound healing in skin (Demaria et al., 2014), senescent stellate cells to tissue repair in liver (Krizhanovsky et al., 2008), and senescent cells play a role in tissue remodeling during embryogenesis (Storer et al., 2013) and parturition (Menon et al., 2016; Menon et al., 2019).

This provides an explanation for the cellular hypertrophy and hypersecretory properties of senescent cells. Working in a coordinate fashion with macrophages (particularly M2 “repair” macrophages), they enact tissue remodelling functions in various contexts, including major and minor tissue trauma. This includes debriding damaged tissue (e.g. breaking down extracellular matrix), preventing fibrosis, promoting re-epithelialization and laying down new extracellular matrix (Godwin et al., 2013; Lucas et al., 2010; Nucera et al., 2011; Plikus et al., 2021; Rajagopalan and Long, 2012).

Given that such tissue-remodeling processes not part of the aging process, the term “cellular senescence” has become something of a misnomer, that is both confusing and obsolete. One proposed solution is to update terminology, for example replacing “cellular senescence” with *remodeling hypertrophy*, “senescent cells” with *remodeling cells* and “senescence-associated secretory phenotype (SASP)”with *remodeling-associated secretory phenotype* (*RASP*) (Gems and Kern, 2022).

These considerations raise questions about the nature of putative senescent chondrocytes observed in OA tissue. Fibroblasts, in which cellular senescence is best characterized, play a similar role in connective tissue to that of chondrocytes in articular cartilage, and fibroblast-like synoviocytes in the synovium: tissue formation and homeostasis, and repair after major trauma (e.g. bone fracture repair) (Marsell and Einhorn, 2011). Moreover, remodeling fibroblasts engaged in wound healing show both SA-β-Gal and p16 expression (Demaria et al., 2014) (as do remodeling macrophages, which risks their being mistaken for senescent cells (Childs et al., 2016; Hall et al., 2016; Hall et al., 2017)). That SA-β-Gal and p16 expression both occur during remodeling hypertrophy raises the possibility that increases in these markers with increasing age in human chondrocytes (Diekman et al., 2018; Martin and Buckwalter, 2003) reflects remodeling hypertrophy (i.e. chondrocyte hypertrophy). Similarities between (putative) chondrocyte senescence and chondrocyte hypertrophy have been previously noted (Rim et al., 2020).

### Developmental changes in chondrocytes in later life

**Figure 4:**
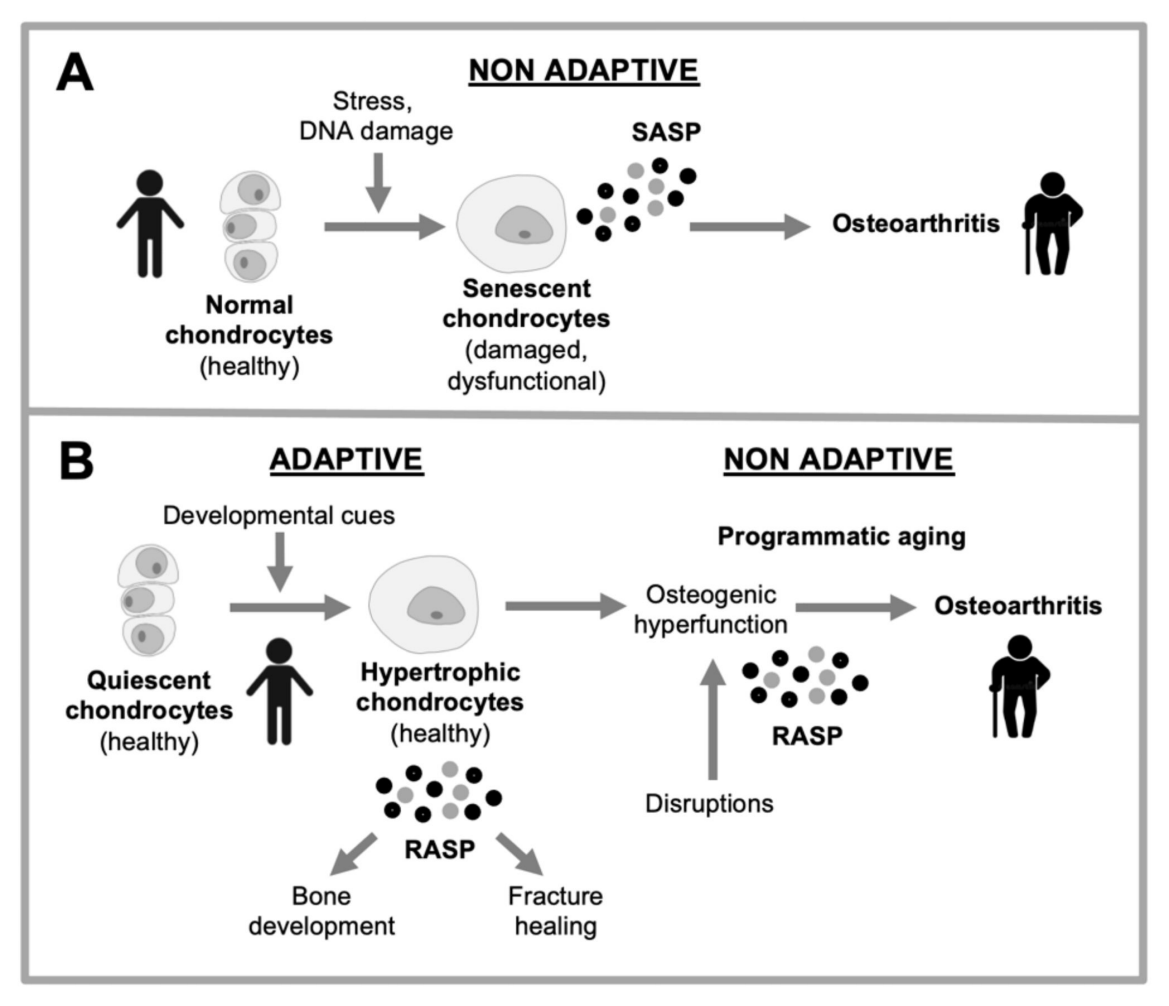
Two explanations for changes in chondrocyte behavior in osteoarthritis. A, Disruption-based explanation. Effects of stressors (e.g. DNA damage, ROS) cause chondrocytes to undergo cellular senescence. Consequent dysfunction leads to hypertrophy and hypersecretion, which damages articular cartilage. B, Programmatic explanation. As part of their normal function in bone development and bone fracture healing, chondrocytes become hypertrophic and hypersecretory. Their remodeling function is executed via the remodeling-associated secretory phenotype (RASP) (Gems and Kern, 2022). Programmatic changes during aging lead to futile activation of chondrocyte remodeling function, leading to transformation of hyaline cartilage into bone, leading to cartilage atrophy and bone hypertrophy.

An alternative view of the age-changes occurring in the cartilage chondrocytes is that they reflect quasi-programmed developmental change, as follows. Chondrocytes develop through a series of stages, first emerging from the differentiation of mesenchymal stem cells (chondroblasts), to perform the task of generating cartilage. They can then undergo hypertrophy, increasing 10-15-fold in volume (Farnum et al., 2002) and become osteogenic, converting cartilage into bone in the process of endochondral ossification. Once their work is done, hypertrophic chondrocytes undergo apoptosis. The formation of articular cartilage requires, in a manner of speaking, that a program for osteogenesis be frozen in mid-stream. One interpretation of the development of OA is that this block fails, leading to run-on into futile osteogenesis (Dreier, 2010; van der Kraan and van den Berg, 2008; van der Kraan and van den Berg, 2012). Thus, plausibly, the age changes in chondrocytes that cause OA are better understood as remodeling hypertrophy that is part of an osteogenic quasi-program than as cellular senescence (Figure 4).

Gene expression changes occurring during chondrocyte hypertrophy include a decrease in hyaline cartilage markers such as aggrecan, collagen type II, and SOX9, and high expression of runt-related transcription factor 2 (RUNX2), matrix metalloproteinase-13 (MMP-13), alkaline phosphatase and collagen type X, a homotrimer-forming short chain collagen. Notably, articular chondrocytes in OA show features characteristic of hypertrophic differentiation, including expression of MMP-13 (Poole et al., 2003), alkaline phosphatase (Pfander et al., 2001), and collagen X (von der Mark et al., 1992). Moreover, OA cartilage typically becomes mineralized (Dreier, 2010; Fuerst et al., 2009), with hypertrophic chondrocytes often co-localized with deposits of calcium pyrophosphate dihydrate crystals and hydroxyapatite (Ishikawa et al., 1989). Taken together, this supports the view that a major driver of OA is an endochondral ossification quasi-program, previously described as “illegitimate hypertrophic differentiation” (Dreier, 2010), involving chondrocyte hypertrophy. This appears to involve not so much an exact recapitulation of normal endochondral ossification, as a muddled attempt at one, with more emphasis on the earlier collagen breakdown stage than the later ossification stage; this is consistent with the axiom that quasi-programs are imprecise by nature (Blagosklonny, 2007).

A critical question here is: what causes the late-life activation of this osteogenic quasi-program? Chondrocyte differentiation is subject to a complex system of autocrine, paracrine and endocrine regulatory control, including factors known to be involved in OA development (Dreier, 2010; Goldring, 2012). Among these are BMP, Wnt and TGF-β signaling, the last which inhibits the progression from cartilage to bone, by acting on the ALK5 (activin-like kinase 5) TGF-β receptor (Ferguson et al., 2000; Yang et al., 2001). Based on this, one suggested OA etiology is loss of inhibition by TGF-β (van der Kraan and van den Berg, 2008).

A possible interpretation here is that loss of this critical off-switch contributes to the loading of the osteoarthritic gun (Figure 2). Secondary factors (including disruptions) can then trigger run-on of a chondrogenic program into an osteogenic quasi-program. Supporting this model, mice in which this TGF-β signal is blocked (by mutation of Smad3) show increased levels of collagen X-expressing cells (hypertrophic chondrocytes) in their articular cartilage, and OA-like joint degeneration, including formation of large osteophytes (Yang et al., 2001).

Why might loss of the TGF-β off switch occur? This can be taken as a question about both evolutionary causes, which are the ultimate reason that aging occurs, and proximate mechanisms of aging. Here it is helpful to consider a suggestion by George Williams for how antagonistic pleiotropy might operate in terms of gene function, which used a hypothetical example involving a gene that promotes calcium deposition into bone. In his scenario, a new mutation in this gene enhances calcium deposition thereby accelerating bone mineralization during development, and promoting fitness; however in later life it increases vascular calcification, promoting pathology (Williams, 1957). He argued that an off-switch for such calcium deposition could in principle evolve and reduce arteriosclerosis; but such a switch could be absent in later life due to the selection shadow (i.e. lack of selection for it).

Employing a similar evolutionary physiology argument, van der Kraan and van den Berg reasoned that the beneficial TGF-β off-switch is not retained in later life due to the selection shadow (van der Kraan and van den Berg, 2008). Wondering about trade-offs that might favor the evolutionary loss of the off switch, they suggested that maintenance of the switch might be costly in resource terms. This is in line with the disposable soma theory of aging, an influential, older alternative to programmatic theory (Kirkwood and Rose, 1991). However, it is not clear why maintenance of signaling should be costly in resource terms. More plausibly, some form of programmatic change, perhaps arising from signaling constraint (Gems and Kern, 2024) leads to loss of the TGF-β off switch.

### Inhibiting the osteogenic quasi-program to prevent osteoarthritis

Hyperfunction of the growth-promoting mammalian Target of Rapamycin (mTOR) pathway plays a prominent role in programmatic aging, and contributes to many late-life diseases (Blagosklonny, 2006; Tsang et al., 2007). The programmatic theory emerged in part from the discovery that inhibition of signaling pathways that promote growth and development can retard aging (Blagosklonny, 2006; de Magalhães and Church, 2005), and the deduction that this particularly involves suppression of hyperfunction and quasi-program expression. Notably, hypertrophy and hypersecretion during cellular senescence is promoted by mTOR, and inhibited by mTOR-inhibitory drugs such as rapamycin (Demidenko et al., 2009). This suggests that mTOR-driven hyperfunction could contribute to OA pathogenesis, and provide a target for preventative intervention.

Human chondrocytes from OA cartilage show elevated mTOR expression, and the same is true for induced OA in mouse and dog models (Zhang et al., 2015). They also show reduced levels of autophagy, a process inhibited by mTOR. While this could in principle reflect cellular senescence, chondrocyte hypertrophy is also promoted by mTOR (Chen and Long, 2014). Notably, bone growth can be inhibited by rapamycin (Chen and Long, 2014; Pal et al., 2015). Moreover in mouse models of OA, disease severity is reduced by both rapamycin and cartilage-specific deletion of mTOR (Carames et al., 2012; Matsuzaki et al., 2014; Takayama et al., 2014; Zhang et al., 2015). Thus pharmacological inhibition of mTOR might inhibit the quasi-programmed endochondral ossification that, as argued here, is the main pathogenetic process in OA in humans; in clinical trials, mTOR inhibitors have been shown to inhibit rheumatoid arthritis (Lin et al., 2022).

About treatment timing: if, as proposed here, OA is a developmental (maturo-developmental) disease, the effective way to treat it will be to pre-emptively block expression of the osteogenic quasi-program. By contrast, treatments that block chondrocyte hypertrophy or kill hypertrophic chondrocytes will have little effect on OA that has already developed. For example, it was recently reported that in marmosets rapamycin treatment from 9.2±3.0 year of age until death (2.1±1.5 years later) did not reduce OA burden; however, the authors noted that many of the test animals already had some degree of OA at the start of the treatment (Minton et al., 2024). In line with this view, a study of late-stage OA, specifically in joints removed during knee replacement surgery, found markers of chondrocyte hypertrophy to be absent (Brew et al., 2010).

What of the role of autophagy in chondrocytes in OA etiology? An apparent decline in autophagy in chondrocytes with age in humans and mice has been linked to chondrocyte cell death, and OA (Lotz and Caramés, 2011). However, for reasons described here, chondrocyte cell death is unlikely to be a cause of OA. In fact, killing all articular chondrocytes using cell-autonomous expression of diphtheria toxin *reduced* injury-induced OA in mice (Zhang et al., 2016), plausibly by pre-empting development of pathogenic hypertrophic chondrocytes.

### A multifactorial model of osteoarthritis

If the multifactorial model (Figure 1) has explanatory value with respect to general mechanisms of aging, then it should be possible to derive a specific version of it for each individual late-life disease. The process of fitting the multiple causes proposed for a given multifactorial disease into an integrated multifactorial model entails generation of diverse new hypotheses and questions about disease etiology.

**Figure 5:**
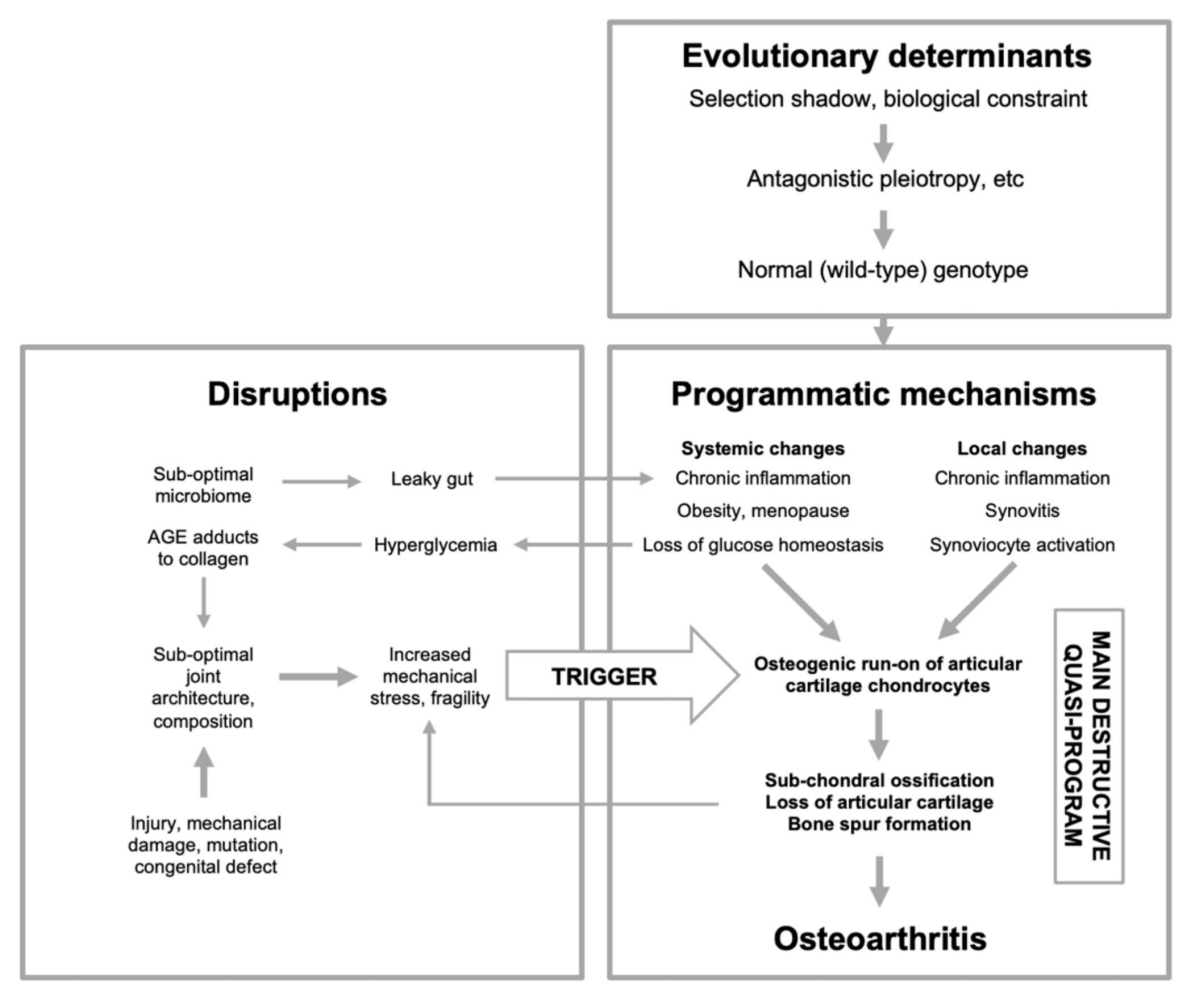
Osteoarthritis etiology viewed in terms of the multifactorial model. Here OA is primarily an evolutionary disease: evolutionary determinants generate a normal (wild-type) genome that specifies programmatic determinants of OA. The main driver of disease is quasi-programs enacted by cells controlling development and tissue-level homeostasis within joints, particularly chondrocytes. Primary programmatic changes create predisposition to OA, creating susceptibility to OA-inducing triggers, caused by disruptions. Changes to joint architecture create further mechanical triggers, in a positive feedback loop. AGE, advanced glycation end products. This is a highly simplified scheme; for example, obesity acts in multiple ways, including increasing load on joints, increasing inflammation which may promote programmatic change in chondrocytes, and increasing type 2 diabetes, hyperglycemia and AGE adducts to cartilage collagens. Menopause is of programmatic origin, and contributes to OA by reducing estrogen levels (Mei et al., 2022).

Figure 5 presents a prototype evolutionary medicine-based representation of OA through the lens of the multifactorial model. According to this scheme, the core mechanisms of OA are specified by the wild-type genome, and expressed in late life in the form of pathogenic quasi-programs. The best characterized is the transition of the chondrogenic developmental pathway in joint cartilage from a useful program for cartilage maintenance into a futile and destructive program of osteogenesis that causes dysfunctional morphogenetic changes to articular joint architecture.

Guided by the standard view of disease as caused by disruptions (Figure 1D), various types of insult have been listed as causes of OA, including mechanical damage (Radin et al., 1991), modification of articular collagen by advanced glycation end products (AGEs) (Liu et al., 2024), and dysbiosis of the microbiome (Arora et al., 2021; Favazzo et al., 2020). While these causes contribute to OA, arguably all are secondary factors that trigger the primary, programmatic disease mechanism; likewise poverty, cold and malnutrition can trigger the development of the active form of tuberculosis, where the primary cause is infection with the bacterium *Mycobacterium tuberculosis*.

In the case of the microbiome for example, leakage of bacterial antigens though the gut into the blood stream may contributes to the development of systemic inflammation (Biver et al., 2019), which as inflammaging is itself, plausibly, a primarily programmatic process contributing to multiple diseases of aging, including OA (Motta et al., 2023). The contributory role of systemic factors in OA is supported by the observation that parabiosis (linkage of circulation) of young and old mice can reduce it in the latter (Li et al., 2020). Similarly, synovitis (inflammation of the synovial membrane) increases levels of inflammatory mediators within the joint (Sellam and Berenbaum, 2010), which may promote programmatic change in chondrocytes.

This account suggests that the primary, programmatic mechanism of OA, involving chondrocyte hypertrophy, may itself be a downstream element of a cascade of triggered quasi-programs (Kern and Stebbing, 2023). If so, then the constraints and trade-offs generating primary pathogenic triggers may be those involved in the origins of chronic, late-life inflammation.

Positive feedback may also contribute to disease progression, as joint dysfunction promotes inflammation and increased tissue repair function, which in turn promote destructive quasi-programs, leading to a vicious cycle. Chondrocyte apoptosis is not a primary cause, but rather a final stage in the osteogenic quasi-program. Accumulation of AGEs in collagen is exacerbated by hyperglycemia resulting from type 2 diabetes, a largely programmatic disease, an example of programmatic molecular damage (Gems and Kern, 2024), contributing to the triggering of the main disease-driving quasi-programs.

Regarding the genetics of OA, several hundred gene variants have been identified that increase disease risk, which in some cases affect Wnt, BMP and TGF-β pathway determinants of chondrocyte development (Zhai and Huang, 2024). In principle, these could affect disruption-type triggers of OA (e.g. by altering joint morphology) or the core programmatic mechanisms (e.g. by altering chondrocyte quasi-programs).

Programmatic theory, as applied here, predicts that a major, primary cause of OA is wild-type genes involved in chondrocyte developmental functions that exhibit antagonistic pleiotropy (AP). Thus, identifying such genes is of particular interest - but also difficult. AP genes are usually identifiable by virtue of allelic variation that alters the balance of benefit and cost; for example, a number genes affecting both immune function and late-life neurological disease risk have been identified in this way (Provenzano and Deleidi, 2021). However, many (most?) AP genes will have evolved to fixation, i.e. lack such allelic variation (Gems and Kern, 2024). Thus, genes whose AP is rendered visible by allelic variation are likely the tip of an iceberg.

A likely example of AP is *GDF5* (growth differentiation factor 5), where a polymorphism linked to OA risk colocalizes with peaks of positive selection (Capellini et al., 2017). It has been suggested that changes to knee morphology linked to the evolution of bipedalism entailed selection for chondrocyte-expressed alleles that, due to biological constraint, exhibit AP and increase OA risk (Richard and Capellini, 2021; Richard et al., 2020).

### How might biological constraint lead to osteoarthritis?

The proposed model (Figure 5) portrays the primary causes of osteoarthritis as programmatic, and specified by the wild-type genome. According to recent theory, biological constraint, particularly of the interconnection type (Figure 1C), leads to programmatic trade-offs that cause disease (Gems and Kern, 2024). Thus, according to the model, to fully understand the programmatic causes of OA will require identification of its causative trade-offs and the constraints that give rise to them. While the nature of such constraints remains little explored, recent theory provides some pointers for hypothesis generation, as follows.

An instructive example of how constraint can give rise to trade-offs, AP and programmatic age-related disease relates to cardiovascular disease. The protein product of the AP gene *ORL1* (lectin-like low-density lipoprotein receptor 1) is thought to promote immunity by binding bacterial cell wall proteins. However, the *ORL1* protein may also promote atherosclerosis by binding oxidized low density lipoprotein (LDL) in endothelial cells (Predazzi et al., 2013). Here AP appears to result from biological constraint: the binding properties of the *ORL1* protein to one target (bacterial cell wall proteins) are inseparable from those to another (oxidized LDL).

The *ORL1* example illustrates how constraints that lead to AP in gene action, and to late-life disease, can particularly evolve where responses to life-threatening challenges earlier in life are concerned (here bacterial infection). Arguably, it is where trade-offs involve surviving life-threatening challenges or avoiding reproductive failure that particularly brutal compromises will be made, and the greatest collateral damage tolerated (in the form of late-life disease) (Gems and Kern, 2024; Provenzano and Deleidi, 2021). Given that articular joints are not reproductive organs, this suggests that constraints causing diseases of aging will often involve those operative in repair of life-threatening tissue injury, such as repair after infection (and immune defense itself) and wound healing, e.g. in skin and bone.

For OA, this suggests the hypothesis that constraints relating to bone fracture healing plays a role in late-life priming for quasi-programmed endochondral ossification. The process of fracture healing to some extent recapitulates developmental endochondral bone formation, including chondrocyte hypertrophy (Bahney et al., 2019; Marsell and Einhorn, 2011). According to this scenario, the quasi-program driving endochondral ossification in OA is less an ontogenetic one than a maturo-developmental, remodeling one (Gems et al., 2024b). Changes during chondrocyte hypertrophy in bone fracture healing are similar to those in osteogenesis (Bahney et al., 2019). A useful question may be whether the changes to chondrocyte function during OA show some features normally restricted to bone fracture healing.

What, then, might be the programmatic mechanisms that cause late-life priming of articular cartilage for quasi-programmed endochondral ossification? One hypothetical possibility is that there exist constraints on the signaling systems regulating immune and tissue remodeling functions. Such constraints could lead to developmental costs, in the form of maladaptive developmental and morphogenetic changes to bone in articular joints. In other words, informational constraints where signaling factors that regulate endochondral ossification) (Dreier, 2010; Goldring, 2012) serve multiple function leads to pathogenic cross-talk, promoting harmful developmental quasi-programs. This may reflect signaling constraints within the joint itself, and/or systemic factors (Figure 5).

The fact that OA is not only widespread among mammals (Föllmi et al., 2007; Greer et al., 1977), but also present in birds (Rothschild and Panza, 2006), reptiles (Isaza et al., 2000; Wolfe et al., 2015) and amphibians (Zhang et al., 2021) suggests the presence of evolutionarily ancient, insuperable constraints in musculoskeletal development and tissue level maintenance. In amphibians and fish, as in higher vertebrates, hypertrophic remodeling (“senescent”) fibroblasts and macrophages work together to effect tissue remodeling during development, and also limb regeneration (Godwin et al., 2013; Yun, 2018).

## Conclusions

An adequate account of the general principles governing the development of late-life diseases should serve a similar role to that of the periodic table in chemistry, that can instruct understanding of the chemical composition of all matter. The multifactorial model-based account of OA presented here (Figure 5) has strengths and weaknesses. To fully understand how senescence generates diseases requires an explanation in terms of evolutionary physiology (Arnold and Rose, 2023). This the programmatic theory component of the multifactorial model provides, which is broadly in line with the developmental model of OA (Dreier, 2010; van der Kraan and van den Berg, 2012). Here OA is a hyperfunctional process, involving chondrocytes that are “activated” (Goldring, 2012), and an endochondral ossification quasi-program.

It is notable here how the multifactorial model offers an explicit account of disease etiology that would be difficult to achieve using the main, earlier evolutionary physiology model, the disposable soma theory (Kirkwood and Rose, 1991). More broadly, the multifactorial model offers a conceptual framework that can help the ambitions of evolutionary medicine to be more fully realized (Brüne and Schiefenhövel, 2019; Nesse and Williams, 1994).

Another strength of the multifactorial model is that it offers a big picture understanding of OA that can incorporate many different existing theories about disease etiology. The great wealth of detail about the molecular and cellular biology of chondrocytes, generated in recent years, risks becoming a surfeit of information such that it becomes difficult to see the forest for the trees. A big picture account allows such findings to be contextualized in a manner that allows their relative importance and significance (position in the forest) to be seen.

However, the presented account is very much a prototype and, at best, to a mature understanding of osteoarthritis what a medieval mapa mundi is to a modern world atlas, with major errors and whole continents missing. But such integrated overviews are necessary, possible and currently lacking, and it is hoped that the Mark I prototype proffered here might provide a starting point for the development of better general models.

In particular, such overviews are critical for generating relevant hypotheses and questions, thereby fruitfully guiding experimental research. These include: how does antagonistic pleiotropy determine OA, and which genes does this involve? How have constraints and the selection shadow led to trade-offs in wild-type function that underpin programmatic OA? And: how is the late-life timing of the programmatic emergence of OA specified? Such questions illustrate how, if the proposed model is realistic, it should at least enable investigators to orient themselves in terms of general principles in a way that allows facts to fall into place, and leads to useful questions to ask.
